# Features of formation of *Yersinia enterocolitica* biofilms

**DOI:** 10.14202/vetworld.2019.136-140

**Published:** 2019-01-25

**Authors:** E. Lenchenko, D. Lozovoy, A. Strizhakov, Yu Vatnikov, V. Byakhova, Eu Kulikov, N. Sturov, V. Kuznetsov, V. Avdotin, V. Grishin

**Affiliations:** 1 Department of Veterinary Medicine, Moscow State University of Food Production, Moscow, Russia; 2 Department of Veterinary Medicine, Agrarian Technological Institute, Peoples’ Friendship University of Russia, Moscow, Russia; 3 Department of General medical practice, Medical Institute, Peoples’ Friendship University of Russia, Moscow, Russia; 4 Department of Agrobiotechnology, Agrarian Technological Institute, Peoples’ Friendship University of Russia, Moscow, Russia

**Keywords:** adhesion, biofilms, clusters, electron microscopy, heteromorphism, matrix, optical microscopy, stereoscopic microscopy, Yersinia

## Abstract

**Aim:**

The work aimed to study the morphology of colonies and their comparison by features of the formation of *Yersinia enterocolitica* biofilms.

**Materials and Methods:**

Bacteria were cultured on a Yersinia Selective Agar medium (“CIN-agar”) at 28°C for 24 h. The microorganisms were grown in meat-peptone broth with 1.0% glucose to measure the absolute values of the optical density of the culture. The optical density of the liquid was determined in a microplate photometric analyzer Immunochem-2100 (HTI, USA) at a wavelength of 490 nm. For the study of biofilms, the specimens were fixed for 3-5 h in pairs of 25.0% solution of glutaraldehyde (according to DV), and pairs of a 1.0% aqueous solution of osmic acid (OSO_4_) were used for contrasting for 2-3 min. The specimens were examined with stereoscopic microscopy “BIOMED MS-1 Stereo” (Russia) and scanning electron microscope “*TM 3030 plus*” (Holland).

**Results:**

With stereoscopic microscopy of the colonies of *Y. enterocolitica*, the S-forms had an elevated intensely colored center, radial striation along the periphery, smooth edges, *d* ≤ 1.0 mm. R-form colonies had a dark color and a dry surface, were tuberous and had a dense center with a peripheral ridge, rugged edges, *d* ≥ 1.5 mm. The optical density of the *Y. enterocolitica* S-form showed that this type of microorganism belongs to the moderate producers of biofilms since the optical density of the sample (density of the sample - Ds) exceeded the optical density of control (density of the control - Dc) by 3 times. In *Y. enterocolitic* a R-form (*D* ≤ 0.197) weakly produced biofilms, the optical density of the sample exceeded the optical density of the control by <2 times.

**Conclusion:**

The ability to form biofilms, the variability of phenotypic features, and the multiplicity of virulence factors of bacteria significantly reduce the effectiveness of diagnostic studies. The development of accelerated methods of detection and differentiation of the virulent properties of pathogenic bacteria will allow scientifically to substantiate and develop a set of measures aimed at preventing animal diseases and obtaining safe livestock products to prevent human diseases. Thus, we need to pay attention to which forms of colonies do *Y. enterocolitic* a form on solid nutrient media: S- or R-forms. Through this study, we know that bacteria-forming S-shaped colonies are more capable of forming biofilms than R-forms. It means that they are more pathogenic and can cause persistent infections due to adhesion and biofilm formation.

## Introduction

Control of the safety of food raw materials of animal origin by bacteriological indicators is an acute problem due to the increase in the number of human diseases caused by toxigenic bacteria, including *Yersinia enterocolitica* [1,2]. It was found that 27.8% – 30.8% of *Y. enterocolitic* a strains isolated from pigs had adhesive properties and the ability to form biofilms, which is related to the hydrophobicity of the cell wall and the presence of virulence plasmids [3]. Of the seven isolates of *Y. enterocolitica* isolated from food samples, a moderate capacity to form biofilm was detected in three strains and the presence of virulence factors in five strains [4]. Biofilms of bacteria *Y. enterocolitica* were detected after repeated treatment, simulating everyday disinfection, which implies the possibility of survival and preservation of the pathogen in the environment [5].

In the light of modern data, biofilms of microorganisms are the community of cells secreting the polymer matrix and adhered to the tissues of the body, abiotic surfaces. They are extremely widespread in natural ecosystems, food products, on instruments and equipment [6-8]. In this regard, to study the measures to control and prevent socially significant infectious diseases, including yersiniosis, research of the interrelationship between biofilm formation and the heterogeneous structure of the bacterial population is the priority direction of scientific research, which determined the relevance of the research topic.

The aims of this study were; To study the morphology of *Y. enterocolitica* bacteria when cultivated on the Yersinia Selective Agar medium; To study structural and functional features of colony formation on solid nutrient medium and biofilm formation by bacteria *Y. enterocolitica*; To compare or find the relationship between these two characteristics.

## Materials and Methods

### Ethical approval

This study was not involved any human or animal subject, and no ethical approval was required.

### Cultivation of colonies

In the experiments, two cultures of microorganism were used: *Y. enterocolitica* S- and R-forms of colonies on solid nutrient media, obtained from the collection of microorganisms from State Research Institute for Standardization and Control of Medical Biological Preparations named after L. A. Tarasevich (Moscow, Russia).

To study the colony morphology, the bacteria were cultivated on a Yersinia Selective Agar medium (CIN agar) at 28°C for 18 h.

Microorganisms were grown in meat-peptone broth (MPB) with 1.0% to measure the absolute values of the optical density of the culture, the optical density of the liquid was determined in a microplate photometric analyzer Immunochem-2100 (HTI, USA) at a wavelength of 490 nm. For this, 200 µl of cultures of microorganisms in a concentration of 105 CFU/ml, diluted 1:100 (experiment), and sterile MPB (control) were added to the wells of 96-well plates. Cultivation was carried out in a thermostat at 37°C for 24 h. The liquid was then removed from the wells, washed 3 times with phosphate-saline solution (pH 7.2), and placed in a thermostat at 60°C for 60 min. To each well, 200 µl of a 0.1% solution of crystal violet was added. After 20 min, the wells were washed 3 times with phosphate-saline solution, dried, and fixed for 30 min in 200 μl 95% ethyl alcohol [3].

### Biofilm formation

For the study of the morphology of biofilms, the coverslips were placed in Petri dishes containing 20.0 ml of MPB; 5.0 ml of a slurry of 18-h cultures of microorganisms (concentration of 105 CFU/ml) was added and cultivated at 37°C. A specimen was fixed for 3-5 h in pairs of 25.0% solution (glutaraldehyde in DV), and pairs of a 1.0% aqueous solution of osmic acid were used for contrasting for 2-3 min [1]. The preparations were studied with stereoscopic microscopy “BIOMED MS-1 Stereo” (Russia) and scanning electronic “Hitachi TM 3030 Tabletop Microscope” (Japan). To obtain representative information, studies were performed by randomly selecting a microscope field of view.

Experiments to determine the optical density were performed in three repetitions, for statistical analysis of the results of the experiments, the criterion of the accuracy of Student’s t-test was used, and the differences were considered statistically significant when p≤0.05.

## Results

**Figure-1:**
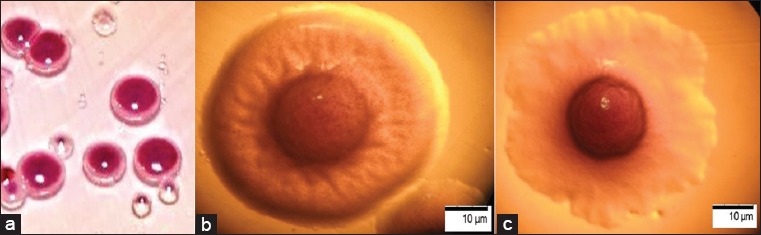
Morphology of bacterial colonies: (a) Growth of colonies on the environment “CIN-agar”; (b) stereoscopic microscopy of S-forms of colonies, 100×; (c) stereoscopic microscopy of R-forms of colonies, 100×.

On the “CIN agar” medium, after 18 h of cultivation, the bacteria *Y. enterocolitica*, fermenting mannitol, formed a pink colony with a clearly defined center and transparent edges (Figure-1a).

With stereoscopic microscopy of the rounded S-form, the colonies had an elevated intensely colored center, radial striation along the periphery, smooth edges, d≤1.0 mm (Figure-1b). R-form colonies had a dark color and a dry surface, were tuberous, and had a dense center with a peripheral ridge, rugged edges, d≥1.5 mm (Figure-1c).

Taking into account the absolute values of the optical density (density, D), it was established that *Y. enterocolitica* S-form (D=0.319) belonged to moderate producers of biofilms since the optical density of the sample (density of the sample - Ds) exceeded the optical density of the control (density of the control - Dc) by 3 times. *Y. enterocolitica* R-form (D≤0.197) belonged to weak producers of biofilms - the optical density of the sample exceeded the optical density of the control by <2 times.

The study of the morphology of biofilms of bacteria in optical and scanning electron microscopy also confirmed the varying degrees of biofilm formation in two species of *Y. enterocolitica* and made it possible to identify the stages: Adhesion; fixation of the monolayer of cells and intercellular matrix; formation of microcolonies; the formation of a mature biofilm; and dispersion. All these stages were more pronounced in the S-forms of *Y. enterocolitica* than in the R-forms, which again confirm the stronger ability to form biofilms. As confirmation of our results, we used electron microscopy. The photographs show that the biofilm formation in stages, in fact, is worse in bacteria with R-shaped colonies.

Adhesion - the primary sedimentation and attachment of bacteria to the surface under the study - was observed after 6-8 h of experimental studies. As a rule, separately located bacteria or several bacterial cells united by a common intercellular matrix were detected. The beginning of structural and functional rearrangements was accompanied by the fact that the cells joined by the intercellular matrix were detected in the form of short and long chains.

**Figure-2:**
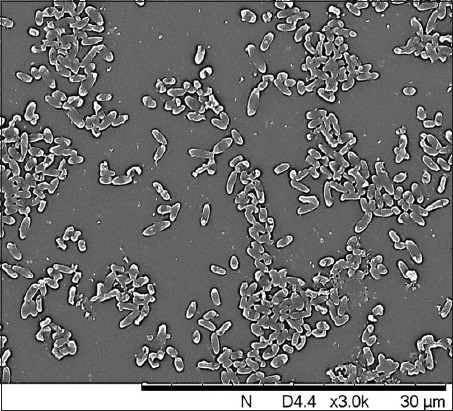
Morphology of bacteria biofilm *Yersinia enterocolitica* S-forms at the stage of fixation, standard error of the mean, 7000×.

Fixation of a monolayer of bacterial cells producing exocellular polymers providing a firm association due to the formation of an intercellular matrix was observed after 18-24 h. As a rule, two types of contacts were observed between the bacteria: Direct interaction, by which clusters are formed, and matrix-mediated interrelations. This indicated that, after the adhesion to the surface is completed, the bacteria actively release the substances that fill the intercellular space. Then, due to the cells attached to the substrate and the intercellular matrix, a monolayer is formed in the form of a diffuse layer on the surface of the test substrate, in our experiments a cover glass. A small part of the bacterial cells was located separately; most cells are located in groups in the form of a monolayer, united by a common intercellular matrix (Figure-2).

The formation of microcolonies, which are the aggregation of bacterial cells, the architectonics of which is supported by the exopolysaccharide matrix, was detected after 24-36 h. Bacteria attached to the surface examined facilitated the attachment of subsequent cells, the intercellular matrix retained the microcolony together, the nutrients accumulated, and the cells began to divide. The conglomerates of cellular structures combined with each other were formed gradually; as the formation of intercellular bonds was formed, the growth of microcolonies was observed. Due to the accumulation of the intercellular matrix, coaggregation of bacterial cells and the formation of a diffuse biofilm layer occurred.

Growth - the formation of a mature biofilm, corresponding to architectonics, and the implementation of intercellular communication processes - was observed after 48-72 h. Cells densely packed and joined by the intercellular matrix attached to the surface were found, which were accompanied by the formation of closed structures of diverse sizes, between which the matrix voids were located. Maintenance of the architectonics of the biofilm is due to intercellular communication of microorganisms, as well as the presence of matrix networks.

Dispersion (ejection of bacteria): As a result of fission, separate cells separate from the biofilm periodically, capable of attaching themselves to the surface after a while, and forming a new colony was detected after 72-96 h. When the intercellular matrix collapses, the peripheric part of the microcolony disintegrates, and microorganism cells are released when the biofilm matrix is dispersed which was accompanied by the death of a part of the cells. Some of the bacterial cells passed to an autonomous existence, which was accompanied by the separation of cells that could attach to the surface and form new colonies. Bacterial cells having an atypical form for the species were arranged in the form of clusters, and the integrity of the biofilm in such areas was disturbed, which was accompanied by an increase in the light refraction. Along with typical rod-shaped cells, cells with a defective cell wall, spheroplasts, protoplasts, L-forms, needle and giant structures, and cell revertants were found.

## Discussion

Analyzing the results and comparing them with the literature data, it should be noted that biofilm is a community of cells attached to the surface and combined with an intercellular polysaccharide matrix, different from plankton bacteria by growth parameters.

When infecting susceptible species, the realization of pathogenic properties of Yersinia is provided by virulence factors, encoded by chromosomal, plasmid genes, and bacteriophages, and integrated into the chromosome; the virulence of pathogenic strains depends on a number of adhesion molecules [9-11].

Adhesions to eukaryotic cells and abiotic surfaces are carried out due to ordered protein molecules, and fine aggregate structures contribute to the formation of a rigid hydrophobic matrix [12-14].

In the dissociation of a homogeneous population into S- and R-forms that differ from random mutations by a high frequency of reversion, the variability of genetic, morphological, physiological, and antigenic properties is observed, and the decrease in metabolic processes and the transition of the population to the “uncultivated state” cause the duration and retrospective of bacteriological studies [1]. S-variants contain all the components of lipopolysaccharides; R-variants express an incomplete polysaccharide; lowering the level of acylation of lipids leads to a decrease in hydrophobicity and an increase in fluidity and the permeability of the outer membrane, which activates the collection, transport, and exchange of lipophilic compounds of cells of susceptible species [12,15].

Changes in surface antigens reduce the effectiveness of serological diagnosis; the inability to extract DNA under standard conditions of lysis causes the complexity of polymerase chain reaction (PCR) diagnostics [16]. The heterogeneity of the population, the transition to a non-cultivated state, indicates the plasticity and variability of microorganisms inherent in the development cycle of the bacterial population, explains the widespread in nature and necessitates the use of enrichment media when isolating a pure culture of microorganisms [1,17,18].

When cells pass into the uncultivated state, a latent persistence of microorganisms arises in the host organism, and cell growth is effective for the growth of non-cultivated microorganisms, direct counting of viable cells by fluorescence, reverse-transcription PCR, flow cytometry, enzyme immunoassay, and recombinant bacteriophage [16].

Exopolysaccharides produced by bacteria provide a protective effect and can slow the diffusion of antibacterial drugs, so the biofilm strains of *Y. enterocolitica* had a pronounced resistance to antibacterial drugs, in comparison with planktonic forms [19,20].

Strategies for the monitoring and control of pathogen biofilms are based on competitive substitution, in particular, lichenisin, produced by *Bacillus licheniformis*, has an antiadherent activity that can prevent and eliminate biofilm formation by pathogenic strains [21]. Potentially useful microorganisms are recommended for water cooling systems [22]. To prevent the formation of biofilms, the methods of the penetration of biocides into the matrix are promising, minimizing the primary contamination and adhesion of bacterial cells is recommended, for example, the effectiveness of acetoacetate and ethyl acetoacetate as inhibitors of *Y. enterocolitica* biofilm has been established [23]. Monoacylglycerols with two specific segments of the fatty acid residue, monolaurin, and monoepherin have strong inhibitory activity against bacterial biofilm *Y. enterocolitica* that is considered a promising method for use in the food industry [24].

## Conclusion

Exopolysaccharides produced by bacteria have a protective effect, which leads to a change in bacterial phenotypic signs, a decrease in metabolic processes, and heterogeneity of the population in the biofilm. The integrity of the cells under the biofilm was disrupted, which was accompanied by an increase in light refraction and a decrease in the optical density. The ability to produce an intercellular matrix with the subsequent formation of a biofilm and the intrapopulation variability of *Y. enterocolitica* expand the survival limits of the species, which allow bacteria to colonize various ecological niches. The ability to form biofilms, the variability of phenotypic features, and the multiplicity of virulence factors of bacteria significantly reduce the effectiveness of diagnostic studies. The development of accelerated methods of detection and differentiation of the virulent properties of pathogenic bacteria, such as the analysis of simple sowing on solid nutrient media, will allow scientifically to substantiate and develop a set of measures aimed at preventing animal diseases and obtaining safe livestock products to prevent human diseases. Therefore, it is very important for specialists to pay attention, what forms of colonies does *Y. enterocolitica* form on solid nutrient media: S- or R-forms. Through this study, we know that bacteria that form S-shaped colonies are more capable of forming biofilms than R-forms. It means that they are more pathogenic and can cause persistent infections due to adhesion and biofilm formation.

## Authors’ Contributions

EL, DL, YV, and EK had the original idea for the study and carried out the design. AS, NS, and VK collected the samples. EK, VA, VG, and VB were responsible for data analysis and data cleaning. EL, YV, VB, and EK drafted the manuscript. The final draft manuscript was revised by all authors. All authors read and approved the final manuscript.
